# Schizophrenia and physical illness: a coordinated care failure

**DOI:** 10.3389/fpsyt.2025.1701118

**Published:** 2025-11-28

**Authors:** Lena G. Larsson, Anna-Karin Olsson, Ann Svensson, Catrin Johansson

**Affiliations:** 1 Department of Health Sciences, University West, Trollhättan, Sweden; 2 Region Västra Götaland, Research, Education, Development and Innovation (REDI), Primary Health Care, Vänersborg, Sweden; 3 Department of Psychiatry, NU-Sjukvården, Trollhättan, Sweden; 4 University West Institutionen for Ekonomi och IT, Trollhättan, Sweden

**Keywords:** collaboration, coordination, mental illness, outpatient psychiatric unit, physical illness, primary care centers, schizophrenia

## Abstract

**Background:**

Patients with schizophrenia have a significantly shorter life expectancy, emphasizing the need for better interventions for physical illness. Limited knowledge, unclear responsibilities, and insufficient collaboration between psychiatric and primary care services increase the risk of inadequate, uncoordinated, and delayed treatment for this vulnerable group. Therefore, the aim of this study was to explore healthcare professionals’ experiences regarding support, treatment, and interprofessional collaboration for patients with schizophrenia and physical illness.

**Method:**

A qualitative study with semi-structured interviews of nine psychiatric and primary care professionals. Data were analyzed using qualitative content analysis.

**Results:**

The analysis generated in an overall theme, Insufficient care coordination for patients with schizophrenia and physical illness of three categories, each with two subcategories. The first category, Inadequate internal clinical protocol, included the subcategories: difficulties in identifying physical illness and differing use of guidelines among healthcare institutions involved. The second category, Deficient division of responsibility included the subcategories: unclear defined division of responsibility for coordinating support and treatment and consequences of shared responsibility for pharmacotherapy. The third category, Lack of common clinical protocols included the subcategories: difficult to get in contact with one another and concrete suggestions concerning common clinical protocols.

**Conclusion:**

To counteract fragmented care for patient group, a more integrated care model is needed. The study highlights the importance of clearer allocation of responsibility, improved communication, standardized routines, and the implementation of coordinated individual care plans (CIP), as well as more user-friendly screening tools to enhance care quality and reduce the risk of treatment errors.

## 1 Introduction

Research has shown that the life expectancy of patients with schizophrenia is 15–20 years shorter than that of the general population (1–3). Moreover, it reveals that this mortality gap appears to be widening (4). The cause of increased mortality among patients with schizophrenia is complex, and it is important that the reservoir of knowledge concerning this issue be expanded (2, 5, 6). Although this patient group is associated with an elevated risk of suicide and accidents, the most common causes of death for patients with schizophrenia are the same as for the general population, i.e. physical diseases such as cardiovascular, infectious, and respiratory system diseases (7–10).

The increased mortality observed within this patient group appears to be linked to physical comorbidities and unhealthy lifestyle choices. According to the World Health Organization (10), high blood pressure, tobacco use, high blood glucose, physical inactivity, and overweight and obesity rank as the leading risk factors for premature mortality in the majority of industrialized countries, and these risk factors are more prevalent within this patient group than they are among the general population (11). Studies investigating the efficacy of interventions aimed at achieving lifestyle changes within this patient group have returned discouraging results: these interventions have neither contributed to increased physical activity nor to improvements related to risk factors for cardiovascular disease (12, 13).

Studies have shown that even when physical diseases in patients with schizophrenia are diagnosed, these patients receive less intensive and lower-quality medical treatment than the general population (14, 15). For example, medication to treat high blood pressure is not prescribed to the same degree to patients with schizophrenia as it is to the general population (16, 17). Moreover, patients with schizophrenia undergo fewer physical examinations and fewer operations and have a lower level of medical treatment compliance and adherence compared to reported averages for patients with the same diseases (18, 19).

The impaired ability of patients with schizophrenia to manage everyday activities, resulting from negative symptoms and cognitive deficits, is widely recognized as a contributing factor to their reduced life expectancy (20). Their reduced cognitive capacity, particularly regarding their ability to remember, initiate, plan, and carry out activities and tasks, can be viewed as a contributing factor in patients with schizophrenia difficulties in managing their physical illness (2). There are also studies that demonstrate that this particular group of patients has a reduced awareness of their own capacity, with the majority overestimating their ability. This too might explain why schizophrenia patients fail to receive the help they need both to seek medical care and to follow up on a planned course of treatment (21, 22). Although there exists an abundance of well-researched interventions for improving these patients’ psychiatric problems, including those intended to prevent their relapse into psychosis and the need for inpatient care, methods for the specialized treatment of patients with co-occurring psychiatric disorders and physical illness remain lacking (23).

Despite the lack of specialized treatment approaches, research has shown that patients receiving pharmacological treatment tend to live healthier lives, experience less stress vulnerability, and are more likely to seek help for their ailments (24–26). Furthermore, the differences in morbidity and mortality rates between patients with schizophrenia and the general population remain pronounced, and addressing this disparity requires interventions at multiple levels. As part of these efforts, patients with schizophrenia should undergo regular examinations for physical conditions to facilitate early detection and management of comorbidities. One example of a protocol-based assessment tool used in this context is the Positive Cardiometabolic Health Resource a structured approach to health monitoring that includes recommended interventions for patients suffering from mental illness who also fall into the cardiometabolic risk group (27). According to Correll et al. (5), integrating mental and physical healthcare in the treatment of this patient group represents a crucial step forward, one that will require concerted policy action and continued research efforts. When Sweden’s National Board of Health and Welfare revised its guidelines (28), it made clear the need to develop supportive measures to assist patients with schizophrenia who also require care for physical illness. To a large extent, patients with schizophrenia have regular contact with outpatient psychiatric units, in particular. More broadly, the healthcare system as a whole is responsible for working to promote healthy lifestyles, and methods need to be developed to prevent the onset of physical illness at patients with schizophrenia an early stage by means of regular examinations, preventive pharmacological therapy, and support in making lifestyle changes (9, 29).

There is insufficient knowledge among, and uncertainty on the part of, healthcare professionals working in physical care regarding patients with severe mental illness (30). In addition, responsibility for coordinating medical interventions on behalf of these patients is often unclear (31). Researchers emphasize the importance of enhanced collaboration between psychiatric and primary care providers to ensure that patients with schizophrenia and co-occurring physical illnesses do not fall through the cracks (31–33).

Although policies and clinical guidelines underscore the importance of coordinated care between psychiatric and somatic care, patients with schizophrenia continue to experience substantial disparities in the management of their physical health. This indicates that existing frameworks are not fully implemented in clinical practice. Moreover, little is known about how healthcare professionals experience and navigate this coordination in their daily work — an understanding that is crucial for developing more effective, person-centered care strategies.

The purpose of this study is to provide an account of healthcare professionals’ experiences regarding support, treatment, and collaboration in connection with patients suffering from both schizophrenia and physical illness.

## 2 Methods

A descriptive qualitative inductive design was applied that made use of semi-structured individual interviews analyzed using qualitative content analysis (34, 35).

### 2.1 Settings and sample

Healthcare professionals involved in patient care and treatment either in outpatient psychiatric care or in primary care centers were invited to participate in this study, using a purposive sampling approach. Information about the study and invitations to participate were initially sent to the managers of the different organizations, who then disseminated them to their healthcare staff members. The study included healthcare professionals whose daily work involves interacting with patients affected by both schizophrenia and physical illness. Any professionals who did not work with patients diagnosed with schizophrenia and physical illness were excluded as informants. The study involved a total of N = 9 healthcare professionals practicing in Sweden, of whom n=5 work at the same outpatient psychiatric care unit and n=4 work at two different primary care centers, all located in close geographic proximity. These nine participants comprised six women and three men who ranged in age from 33 to 60 years. The length of their professional experience varied between eight and 30 years. The professions they represented were as follows: general practitioners and psychiatrists (n=3), occupational therapists (n=1), nurses (n=3), assistant nurses/nursing assistants (n=1), and social workers (n=1). The professionals working at the outpatient psychiatric unit meet patients with varying psychiatric diagnoses in their work at the clinic. The professionals working at the primary care centers meet all kinds of patients suffering from physical illness who visit their center. Prior to the commencement of the study, the researchers had limited knowledge regarding how collaboration was structured and operated between the organizations, in relation to the patient population included in this study.

### 2.2 Data collection and analysis

Interviews with the study’s informants were conducted between April and November 2022. These interviews focused on the following: 1) these professionals’ estimates of their institution’s management of patients with schizophrenia and physical illness, 2) their current approaches and routines for addressing the physical health needs of these patients, 3) the availability of specific support, and 4) how well the collaboration between psychiatric care and primary care functions for this patient group. These digital interviews were recorded and subsequently saved as audio files. The contents of these files was then transcribed verbatim. The interviews ranged from 30 to 64 minutes (md=46 min.) in duration. The data were analyzed using inductive content analysis, in which units of meaning were identified, condensed, abstracted, and coded based on similarities and differences (34). The authors (LGL, AKO and CJ) first read the interviews several times. Thereafter, in one interview, meaning units were identified jointly, condensed, and coded. The remaining interviews were analyzed individually in a similar manner, with regular discussions and check-ins. The codes were then reviewed and abstracted jointly. This process resulted in the formation of subcategories that were organized into categories, and which ultimately gave rise to an overall theme. In the initial phase of the analysis, the descriptions remained closely tied to the original text. However, as the analysis progressed, the level of interpretation increased, leading to the development of subcategories, categories, and the overall theme. An example of the analytical process is presented in **Table 1**.

**Table 1:** Analytical process.

Overall theme: Insufficient care coordination for patients with schizophrenia and physical illness				
Meaning unit	Condensed meaning unit	Code	Subcategory	Category
“Our psychiatric work is systematic, whereas our physical care operates on a more individual level; we don’t take a systematic approach to it.” (Interview 9)	Our psychiatric work is systematic, whereas our physical care operates more on the level of the individual; we don’t take a systematic approach to it.	Identify physical illness	Difficulties distinguishing physical illness	Inadequate internal clinical protocol
“If there’s an established illness, then the boundaries are quite clearly demarcated, but if there isn't, then it’s … In reality, there are other factors that one needs to get to the bottom of, but there’s, like, no [clear path]. In any case, I can’t claim that primary care centres will take charge of [the issue], since we don’t feel that this group is our responsibility, I guess. We’re a bit more like advisors, and we feel that primary responsibility lies with psychiatric care unit, and we monitor an established illness.” (Interview 4)	If there’s an established illness, then the boundaries are clearly demarcated, but if there isn’t … There are other factors that one needs to get to the bottom of, but there’s, like, no [clear path]. I can’t claim that primary care centres will take charge of [the issue], since we don’t feel that this group is our responsibility, I guess. We’re a bit more like advisors, and we feel that primary responsibility lies with psychiatric care unit, and we follow an established illness	Poorly defined division of responsibility	Unclear defined division of responsibility for coordinating support and treatment	Deficient division of responsibility
“Contact between psychiatric care unit and primary care centres isn’t all it could be; it could work better for this group.” (Interview 2)	Contact between psychiatric care unit and primary care centres isn’t all it could be …	Avenues of contact	Difficult to get in contact with one another	Lack of common clinical protocols

### 2.3 Ethical considerations

Participation in the study was voluntary. The study involved healthcare professionals, and no patients participated. Informants received both written and verbal information about the study, including information on their rights as informants. The study adhered to the ethical guidelines stipulated in the Declaration of Helsinki (36), and was approved by the Swedish Ethical Review Authority (reference no. 2021-04875).

## 3 Results

**Figure 1:**
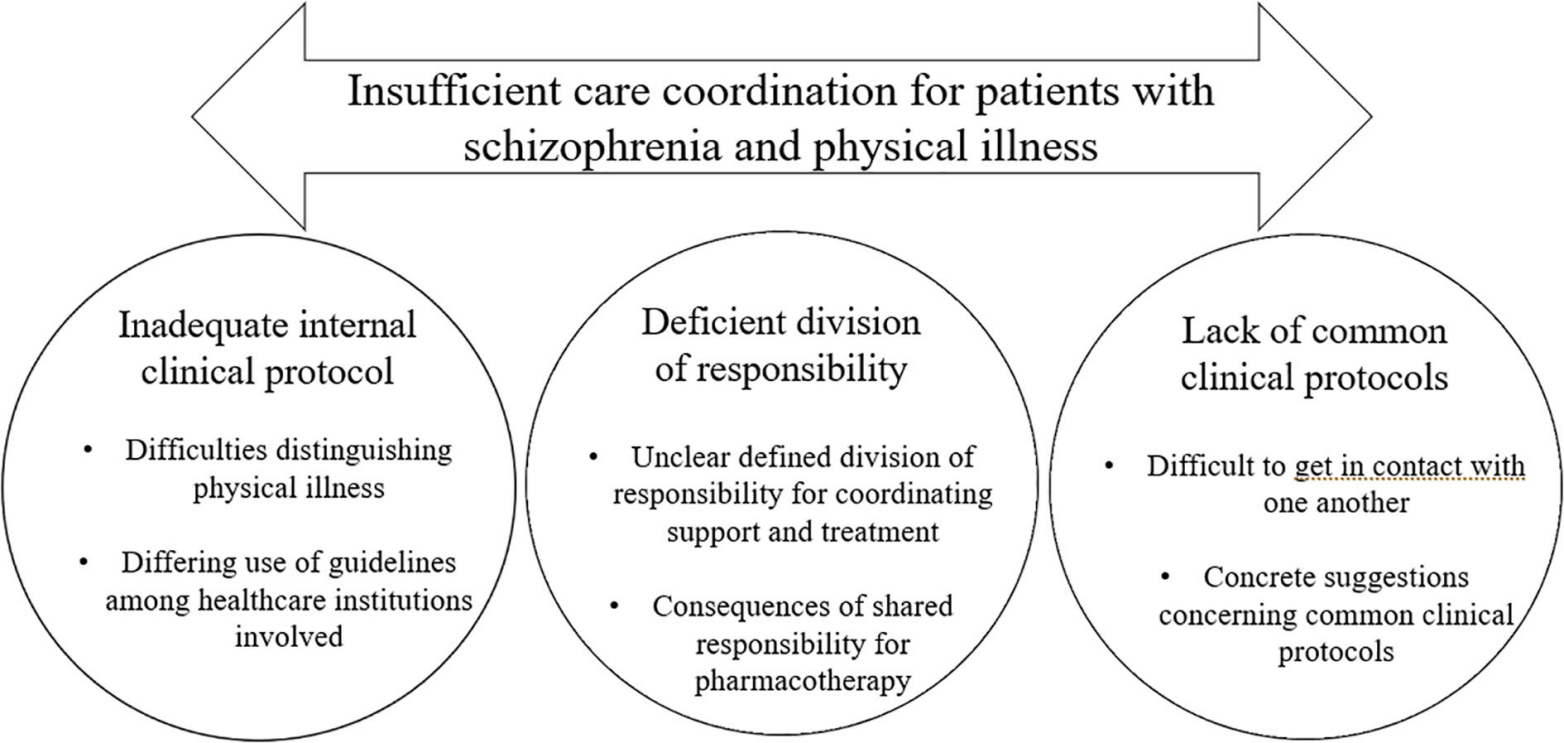
Illustration of the results showing theme, categories, and subcategories.

The analysis of healthcare professionals’ experiences of providing support and treatment for, and of collaborating with other healthcare providers regarding patients with schizophrenia and physical illness yielded an overall theme: *Insufficient care coordination for patients with schizophrenia and physical illness*. This overall theme included three categories, each with two subcategories (**Figure 1**).

### 3.1 Inadequate internal clinical protocol

The first category concerned an inadequate internal clinical protocol, and included two subcategories: 1) difficulties distinguishing physical illness, and 2) the differing use of guidelines among the healthcare institutions involved.

#### 3.1.1 Difficulties distinguishing physical illness

The study’s informants pointed out that a patient’s physical health problems are rarely considered by the outpatient psychiatric care unit, and that healthcare professionals at the primary care centers have difficulty distinguishing physical health conditions from mental complaints. When patients with schizophrenia need to contact their primary care center, healthcare professionals at the psychiatric care unit assess whether the patient is capable of doing this independently, or whether they should assist them by making telephone calls on their behalf or sending referrals.

… I write a referral myself, or I say to our doctor: ‘I’ve observed X or Y. Shall I write a referral, or will you write a referral?’ I also make phone calls from time to time. (Interview 1, outpatient psychiatric unit)

When family members or healthcare professionals from municipal agencies or the psychiatric care unit participated in patients’ with schizophrenia appointments at primary care centers, they were able to contribute valuable information that the patients themselves had difficulty conveying. Even so, these patients risked being referred back to psychiatric care in cases where their symptoms were interpreted as being primarily caused by their mental health issues.

… it’s not easy for these patients to get a foothold in [primary care centers], and they are often told that their ailments likely have psychiatric roots and that they should visit their clinic. So it’s difficult for them to be taken seriously. (Interview 2, outpatient psychiatric unit)

#### 3.1.2 Differing use of guidelines among healthcare institutions involved

With regard to guidelines and protocols for addressing physical health conditions, the study’s informants working at the psychiatric care unit agreed that checklists exist, but had differing opinions concerning to what extent these are used in practice. Some informants maintained that annual physical examinations based on regional medical guidelines [Swedish: *regional medicinsk riktlinje (RMR)*] are performed on all patients, while others believed that such examinations are only performed on patients participating in an on-going research project.

… they’re members of a risk group that’s susceptible to a lot of things, so we do annual health examinations and screen for certain physical ailments. (Interview 2, outpatient psychiatric unit)

All patients with schizophrenia registered at the outpatient psychiatric care unit were offered the opportunity to participate in a patient health education initiative with a focus on diet, physical activity, and sleep. The primary care centers involved in the study had no initiatives specifically targeting this patient group with regard to lifestyle changes. The informants agreed that encouragement and motivational conversations alone were not sufficient to generate improvement among patients with schizophrenia. Moreover, interventions were believed to be more effective when implemented by the mental healthcare professionals, who are both known to and qualified to treat this particular patient group.

… the fact that the patients get help and support from staff who know them and who are actually specially trained to work with this particular patient group. (Interview 3, primary care centers)

### 3.2 Deficient division of responsibility

The second category concerned the deficient division of responsibility and included two subcategories: 1) the unclear defined division of responsibility for coordinating support and treatment, and 2) the consequences of shared responsibility for pharmacotherapy.

#### 3.2.1 Unclear defined division of responsibility for coordinating support and treatment

Uncertainty was apparent among the healthcare professionals from both types of care institution as to who was responsible for coordinating care for patients with schizophrenia and physical illness. All of the informants interviewed felt that the institution with which the patient has most contact should assume responsibility for coordinating their care, and that the other institution should make itself available to provide support and advice.

… the responsibility should lie where the patient is, and then our job is to get involved where we can provide help in the form of advice and support. (Interview 4, primary care centers)

The informants described that when a patient had a confirmed diagnosis, whether psychiatric or physical, the boundaries of responsibility for their care were clearly demarcated: anything related to the patient’s schizophrenia diagnosis was the psychiatric care unit responsibility, whereas support and treatment concerning physical diseases were the purview of the patient’s primary care center. The division of responsibility was also clearly defined with regard to the prescription of assistive technologies: responsibility for cognitive assistive technologies lay with psychiatric care, while assistive technologies for alleviating impaired physical function were the responsibility of municipal agencies or the primary care provider’s rehabilitation unit.

… we treat what’s related to the psychiatric bit of the psychotic disorder, and they’re responsible for what concerns the body. I think that’s their understanding too. (Interview 9, outpatient psychiatric unit)

Confusion regarding the division of responsibility for a patient’s care could arise when patients formally referred to their primary care center by the psychiatric care unit failed to attend their appointment. The study’s informants working at the psychiatric care unit emphasized the need to take an active approach to getting patients to attend scheduled appointments, but did not consider it their responsibility to ensure that patients visit their primary care center. Informants working at the primary care centers related that they handle the patients with schizophrenia in the same manner as they do other patients, which entails both positive and negative consequences for this group. For example, patients are expected to take responsibility for their own healthcare and to contact the center when needed or when the time arrives for scheduled interactions.

… those of us who work in primary care centers don’t feel that this group is our responsibility, I guess. We’re a bit more like advisors, and we feel that primary responsibility lies with psychiatric care, and we just monitor an established illness. (Interview 4, primary care centers)

#### 3.2.2 Consequences of shared responsibility for pharmacotherapy

Responsibility for pharmacological therapies used to treat members of the patients with schizophrenia and physical illness is often shared among a number of prescribers operating independently of one another. Informants working at the primary care centers explained that they are reluctant to alter prescriptions written by psychiatrists. In some cases, it is not possible to change medications despite the fact that the patient’s physical health is negatively impacted by the drug’s side effects, since there is a risk that the underlying disease, their psychiatric condition, will worsen as a result.

… to what extent should one question the psychiatric pharmacological therapy[]? (Interview 3, primary care centers)

The study’s informants also drew attention to an issue related to patients’ failure to take prescribed medication. For instance, a patient might have medication prescribed to them, but then fail to have the prescription filled. The informants felt that it is unclear just what the monitoring of a patient’s pharmacological therapy regimen should include and when it should occur as well as how it should be carried out and by whom. Furthermore, it came to light that no joint evaluations are undertaken that include psychiatrists, general practitioners, and healthcare professionals from the domiciliary care service (for example) when patients with schizophrenia develop symptoms indicative of physical illness.

… I guess we don’t really take responsibility for follow-ups, and, besides, we don’t really know what their diagnoses are. (Interview 6, primary care centers)

### 3.3 Lack of common clinical protocols

The third category concerned the lack of common clinical protocols and included two subcategories: 1) difficult to get in contact with one another, and 2) concrete suggestions concerning common clinical protocols.

#### 3.3.1 Difficult to get in contact with one another

Informants from both types of institution described difficulties in contacting one another. There were no direct phone numbers to call, leading to information and referrals being sent via mail, which prolonged the time needed to process them. Furthermore, it became evident that the healthcare professionals involved did not know one another, and there was a lack of collegial sentiment across institutional boundaries despite the fact that the institutions were housed in the same premises. In cases where a patient had previously been assigned a care coordinator in connection with a physical diagnosis, such as diabetes, it was easier to reach a specific member of healthcare professionals at their primary care center.

… Yes, it’s not all that easy, but one could look at it from the opposite point of view too: How accessible are we here at our primary care center? We don’t have any direct phone numbers. (Interview 5, primary care centers)

#### 3.3.2 Concrete suggestions concerning common clinical protocols

Collaboration between the psychiatric care and primary care institutions has been in a state of decline, and the study’s informants requested a more structured protocol with regard to addressing physical illness for this patient group. They described that the planning of patients with schizophrenia and physical illness treatment was only ever carried out separately within each respective institution. Consequently, following patients’ appointments, it was left up to the patient themselves to convey the resulting information from one institution to the other. Moreover, it was unusual for anyone to convene a joint meeting with regard to a Coordinated Individual Plan (Swedish: *samordnad individuell plan (SIP)*) when patients with schizophrenia and physical illness was in need of coordinated interventions, which was highlighted as an area for improvement.

… it’s a dilemma, really; I don’t always get to know what’s transpired at the primary care center. (Interview 1, outpatient psychiatric unit)

The informants drew attention to the importance of meeting together in facilitating assessments of patient needs and determining which interventions should be offered and where. They considered it important to develop individual solutions for each patients with schizophrenia and physical illness, but doing so requires coordination. One example that illustrates this point are instances where patients did not wish to visit their primary care center, and could instead receive their treatment as prescribed by the primary care provider at the psychiatric care unit instead. The study’s informants believed that greater collaboration is needed to effect change and, in this connection, they also called attention to the importance of collaborating with municipal agencies.

… collaboration between myself and the primary care center is fairly limited, on the whole (Interview 7, outpatient psychiatric unit)

Additional suggestions for improving clinical protocols included creating greater opportunities for consultation and, above all, the request that an individual with a coordinator’s function be appointed for the patients with schizophrenia and physical illness within each institution. The informants also suggested that they could invite one another to join each other’s professional meetings, for example, to discuss shared clinical protocols.

… to hold a staff meeting together with the primary care center where we discuss our collaboration. (Interview 7, outpatient psychiatric unit)

The informants requested the opportunity to increase their knowledge of collaborative practices to enable them to develop common clinical protocols. They also requested training in the use of various assessment tools to help identify and distinguish between this patient group’s mental and physical health conditions.

… we’ll have to try and get up to speed with new findings, since research is ongoing into how we can best help these patients early on. [Such interventions] might cover medication, lifestyle, and support. (Interview 2, outpatient psychiatric unit)

#### 3.3.3 Insufficient care coordination for patients with schizophrenia and physical illness

The study’s overall theme revealed a collaborative arrangement impeded by fragmentation, in which the institutions involved in the study often worked in isolation, and which tended to lack a coordinated, holistic approach. The prevailing division of responsibility for coordinated care for patients with schizophrenia and physical illness was felt to be unclear, and the need for clearer organization and to include municipal stakeholders when coordinating care was emphasized. Patients with schizophrenia and physical illness complaints risked being dismissed as symptoms of their mental illness, which at times led to their erroneous re-referral to psychiatric care. While their opinions differed as to the application of current guidelines, the study’s informants agreed that encouragement and motivational conversations alone are insufficient support to achieve lifestyle changes among this patient group.

## 4 Discussion

The results of this study reveal a significant lack of coordination in the care provided to patients with schizophrenia and physical illness. Our findings are in line with those of Kohn et al. (37), who also describe a systematic division between the treatment of mental and physical illness within the healthcare system.

One key issue that emerged in this study was inadequate internal clinical protocols and their impact on patients’ support and treatment. Additionally, the results reveal challenges associated with identifying and managing physical illness that arise within the schizophrenia patient group in spite of its small size. This finding aligns with that of Jønsson et al. (38), who concluded that general practitioners experience difficulties in providing physical illness to patients suffering from severe mental health issues, due in part to a lack of time and to these patients’ complex health profiles. One possible solution discussed within both literature published in the field and the present study is the use of screening tools and clinical protocols. Positive Cardiometabolic Health Resource is one tool that has proven to be important in identifying risk factors and providing support for recommendations. In healthcare professional’s experience, screening tools used to detect physical health conditions in patients with schizophrenia and physical illness are difficult to implement in practice. This suggests that future research should focus on developing tools that are both user-friendly and not excessively time-consuming (39).

Another important finding from our study concerned the unclear defined division of responsibility for care coordination between psychiatric care unit and primary care centers. Their shared responsibility for a patient’s pharmacological therapy was especially problematic, since it was unclear which of the two institutions was primarily responsible for the follow-up. Collaboration between these two types of institution can be illustrated as a series of levels ranging from separation, coordination, collaboration to complete integration (40, 41). This study can be viewed as exemplifying separation between these institutions as characterized by an expectation that the other party would assume responsibility, with the result that neither one assumed full responsibility for patients’ overall health. This phenomenon is confirmed by previous research (33), which identified the absence of a clearly defined division of responsibility as leading to gaps in care. Studies have shown that, regardless of which organization model is used, it is possible to ensure continuity in healthcare through effective resource coordination among organizations (42).

In addition to a unclear defined allocation of responsibilities, the present study’s results also revealed a general lack of shared protocols and organized forms of collaboration between psychiatric care unit and primary care centers. Earlier research underscores the importance of clear guidelines for multiprofessional collaboration, and that limitations in training and collaboration, time constraints, and organizational barriers negatively impact healthcare providers’ ability to implement an integrated approach to care (37, 43). A review, consistent with our findings, identifies ambiguous delineation of responsibilities, insufficiently structured communication processes, and limited resources as principal barriers to integrated care. Conversely, it emphasizes that clearly articulated role descriptions and shared protocols constitute important facilitators (44). Another review demonstrates that enhanced collaboration between healthcare providers can improve access to integrated care for individuals with schizophrenia and co-morbid somatic conditions; however, only approximately 31% of this patient group in the United Kingdom engage with primary care services. Despite this, the review reported no definitive advantages of integrated care over standard care with respect to primary outcome measures, including quality of life, mental state, and rates of psychiatric admission. Nevertheless, the absence of documented adverse effects underscores the need for further research into the implementation and optimization of integrated care (45).

Sweden’s national guidelines for healthcare providers also emphasize the importance of psychiatric care professional’s active collaboration with primary care providers to ensure that patients receive adequate treatment for both their mental and physical health issues (46).

One recommendation for improvement in this area was to intensify the use of Coordinated Individual Plans, which were introduced in Sweden as far back as 2010 as a means of strengthening collaboration between the healthcare system and Social Services. However, despite healthcare professionals’ good intentions, studies have shown that these plans are not used in an organized manner, and that they tend to shift the focus from improved collaboration to controversies surrounding responsibility and protocols.

### 4.1 Practical implications

Based on the study findings, the following recommendations for best practice are proposed; 1) Strengthen the implementation of coordinated individual care plans through clearer structures, defined responsibilities, and systematic follow-up routines. 2) Ensure an active patient and family perspective in planning and evaluation of care interventions. 3) Develop joint training initiatives and collaboration arenas for different professional groups to promote a shared understanding of goals, roles, and communication channels. 4) Prioritize integrated care models that balance professional expertise, organizational requirements and needs, for patients with schizophrenia and concomitant physical illness.

Furthermore, studies have noted that the patient’s own role is often overlooked, especially when the various professions involved in their care do not share a common understanding (47–51). To overcome these obstacles, future research should focus on developing a more functional collaboration model in which professional experience, organizational needs, and patient perspectives are integrated in a more balanced way for patients with schizophrenia and physical illness (32, 50).

### 4.2 Strengths and limitations

The present study’s modest number of informants (N = 9) and its region-specific sample may be considered limitations, which affects transferability. The lack of any greater response may be explained by the fact that healthcare organizations are currently experiencing significant financial strain during a period of economic cutbacks. However, despite its small sample size, it remains a strength of this study that its informants represented both outpatient psychiatric unit and primary care centers. Another of its strengths is the diversity of professional roles among these informants: general practitioners, psychiatrists, occupational therapists, social workers, nurses, and assistant nurses (52). This diversity likely provided useful variation in the data, which aligns well with one of the aims of qualitative content analysis (34). The potential self-selection bias, where informants who were more interested in collaboration may have been more likely to participate, is acknowledged as a limitation of the study. Efforts were made to encourage broad participation, but the possibility that the sample may overrepresent more motivated individuals cannot be ruled out. The analysis was thoroughly discussed among the authors to achieve consensus, which further enhances its trustworthiness (53). The study’s three authors (LGL, AKO, CJ) have experience in the professional fields represented in the study, namely psychiatric care units and primary care centers. This professional background may have influenced the interpretation of the results, and these interpretations were continuously discussed and critically reflected upon within the research team to enhance reflexivity and transparency.

The analysis was both descriptive and interpretive descriptive at the category and subcategory levels, and interpretive at the theme level. Quotes were used as a means to include the participants’ own statements, and thereby enhance the study’s credibility (53). Although the transferability of the study’s results is another of its limitations, its results ought to be applicable in a comparable context (35).

## 5 Conclusion

The results of this study demonstrate that ambiguously defined roles, insufficient communication structures, and the absence of shared protocols between psychiatric care and primary care contribute to fragmented care for patients with schizophrenia and co-occurring physical illness. Improving care quality requires clearer role delineation within care coordination, systematic implementation of coordinated individual care plans, and more efficient information exchange across services. The development of practical, user-friendly screening procedures, alongside enhanced awareness of integrated care models, is essential. A more coherent and integrated care model is likely to improve patient outcomes while reducing the risk of care omissions and inefficient use of healthcare resources.

## Data Availability

The raw data supporting the conclusions of this article will be made available by the authors, without undue reservation.
